# A88 UNDERSTANDING NURSE PERCEPTIONS OF CARING FOR PATIENTS WITH ALCOHOL USE DISORDER: A CROSS-SECTIONAL STUDY

**DOI:** 10.1093/jcag/gwab049.087

**Published:** 2022-02-21

**Authors:** A Hyde, E Johnson, C Bray, T Meier, M Carbonneau, J Spiers, P Tandon

**Affiliations:** 1 University of Alberta Faculty of Nursing, Edmonton, AB, Canada; 2 University of Alberta Faculty of Medicine & Dentistry, Edmonton, AB, Canada; 3 Cirrhosis Care Clinic, University of Alberta Hospital, Edmonton, AB, Canada

## Abstract

**Background:**

Alcohol Use Disorder (AUD), the problematic consumption of alcohol, affects 107 million people worldwide. AUD increases morbidity and mortality and has a substantial impact on daily functioning including quality of life, relationships and employment. AUD is particularly detrimental in patients who already have liver damage like cirrhosis. The management of AUD includes screening, brief intervention and referral to treatment for psychological and pharmacotherapy based treatment. People with AUD have frequent interactions with the healthcare system. These interactions represent opportunities to engage patients with therapy. As front-line workers who have maximal contact with patients, nurses practicing in acute care are in an ideal position to initiate AUD related discussion with patients. Prior to the design of an educational intervention to increase nursing engagement with AUD screening and brief intervention, there is a need to understand baseline knowledge, attitudes and perceptions in this group.

**Aims:**

The aim of the present study was to explore the knowledge, attitudes and perceptions of nurses caring for patients with cirrhosis and AUD.

**Methods:**

We conducted a cross-sectional survey using the Survey of Attitudes and Perceptions (SAP). The SAP is derived from a validated tool to assess attitudes and perceptions towards patients with AUD. Anonymous surveys were distributed on inpatient medicine units across 5 geographic zones in Alberta between September 2019-March 2020. Data were analyzed using descriptive and inferential statistics.

**Results:**

A total of 93 nurses from 7 inpatient medicine units across Alberta participated in the study. The majority of participants were Registered Nurses (74.9%), who practiced in an urban setting (69%), and had worked in their role for an average of 9.9 years. Few (22.6%) participants reported any prior structured education on caring for patients with AUD, with the majority reporting limited knowledge of alcohol and effects of alcohol consumption. Though most reported that caring for patients with AUD was part of their professional role, only 15.7% felt motivated to work with this group of patients. Responses to individual questions or sub-domains of the survey did not significantly differ by length or time in professional role, or practice setting.

**Conclusions:**

Our results indicate that nurses have limited knowledge on caring for patients with AUD. Given the importance of AUD in the development and progression of cirrhosis as well as the frequency of hospitalizations for patients with cirrhosis, increasing nurse knowledge of AUD is crucial to improving the quality of care for these patients. The results of this study will be used to inform the development of an educational intervention to increase nursing knowledge of caring for patients with cirrhosis and AUD.

**Funding Agencies:**

Alberta Innovates

